# Diurnal secretion of growth hormone, cortisol, and dehydroepiandrosterone in pre- and perimenopausal women with active rheumatoid arthritis: a pilot case-control study

**DOI:** 10.1186/ar2271

**Published:** 2007-07-28

**Authors:** Marc R Blackman, Ranganath Muniyappa, Mildred Wilson, Barbara E Moquin, Howard L Baldwin, Kelli A Wong, Christopher Snyder, Michael Magalnick, Shaan Alli, James Reynolds, Seth M Steinberg, Raphaela Goldbach-Mansky

**Affiliations:** 1 Endocrine Section, Laboratory of Clinical Investigation, National Center for Complementary and Alternative Medicine, National Institutes of Health, 9000 Rockville Pike, Bethesda, MD 20892, USA; 2 Office of the Clinical Director, National Institute of Arthritis and Musculoskeletal and Skin Diseases, National Institutes of Health, 9000 Rockville Pike, Bethesda, MD 20892, USA; 3 Department of Radiology, Warren Magnuson Clinical Center, National Institutes of Health, 9000 Rockville Pike, Bethesda, MD 20892, USA; 4 Biostatistics and Data Management Section, Center for Cancer Research, National Cancer Institute, National Institutes of Health, 9000 Rockville Pike, Bethesda, MD 20892, USA

## Abstract

Rheumatoid arthritis (RA) is associated with neuroendocrine and immunologic dysfunction leading to rheumatoid cachexia. Although excess proinflammatory cytokines can decrease somatotropic axis activity, little is known about the effects of RA on growth hormone/insulin-like growth factor-1 (GH/IGF-I) axis function. We tested the hypothesis that patients with active RA exhibit decreased GH/IGF-I axis activity. To do so, we conducted a pilot case-control study at a clinical research center in 7 pre- and perimenopausal women with active RA and 10 age- and body mass index-matched healthy women. Participants underwent blood sampling every 20 minutes for 24 hours (8 a.m. to 8 a.m.), and sera were assayed for GH, cortisol, and dehydroepiandrosterone (DHEA). Sera obtained after overnight fasting were assayed for IGF-I, IGF-binding protein (IGFBP)-1, IGFBP-3, C-reactive protein (CRP), interleukin-6 (IL-6), glucose, insulin, and lipids. Body composition and bone mineral density were evaluated by DEXA (dual emission x-ray absorptiometry) scans. In patients with RA, mean disease duration was 7.6 ± 6.8 years, and erythrocyte sedimentation rate, CRP, and IL-6 were elevated. GH half-life was shorter than in control subjects (*p *= 0.0037), with no other significant group differences in GH deconvolution parameters or approximate entropy scores. IGF-I (*p *= 0.05) and IGFBP-3 (*p *= 0.058) were lower, whereas IGFBP-1 tended to be higher (*p *= 0.066), in patients with RA, with nonsignificantly increased 24-hour total GH production rates. There were no significant group differences in cortisol or DHEA secretion. Lean body mass was lower in patients with RA (*p *= 0.019), particularly in the legs (*p *= 0.01). Women with active RA exhibit a trend toward GH insensitivity and relatively diminished diurnal cortisol and DHEA secretion for their state of inflammation. Whether these changes contribute to rheumatoid cachexia remains to be determined.

NCT00034060.

## Introduction

Rheumatoid arthritis (RA) is a chronic, autoimmune-mediated, inflammatory arthritis that occurs in approximately 0.5% to 1% of the general population and affects women 2.5 times more often than it does men. Chronic imbalance among neuroendocrine, immunologic, and microvascular systems leads to 'rheumatoid cachexia,' accelerated cardiovascular disease, and enhanced mortality in patients with RA [1-3]. RA cachexia is manifested by losses of muscle and bone mass, resulting in part from augmented cytokine activity [4].

Progressive decline in the secretion of growth hormone (GH) and its principal circulating and tissue mediator, insulin-like growth factor-1 (IGF-I), is one of the key pathophysiological mechanisms contributing to the cachexia of normal aging [5]. In a mouse model, overexpression of the inflammatory cytokine, interleukin-6 (IL-6), has been associated with suppression of the GH/IGF-I axis [6]. However, few studies have investigated the GH/IGF-I axis in patients with active RA [7]. The hypothalamic-pituitary-adrenal (HPA) axis is also affected to varying degrees in patients with RA, independent of the use of exogenous glucocorticoids. Most reports indicate that circulating levels of cortisol and dehydroepiandrosterone (DHEA) are normal, and not elevated, in the setting of increased proinflammatory activity, suggesting a relative hypoadrenalism in patients with RA, possibly due to reduced corticotropin-releasing hormone (CRH) activity [8,9].

We hypothesized that the excess of systemically released inflammatory cytokines characteristic of patients with active RA suppresses GH/IGF-I axis activity and that the combined effects of disordered endocrine (anabolic balance) and immune function contribute to changes in body composition predisposing patients with RA to sarcopenia, increased body fat, and osteopenia. The primary goal of this study was to determine whether spontaneous, diurnal GH secretion and a.m serum IGF-I concentrations are decreased in pre- and perimenopausal women with active RA. In addition, we evaluated ultradian and pulsatile cortisol and DHEA secretory dynamics, body composition, and metabolic outcomes in these same patients and compared them with values in healthy control subjects.

## Materials and methods

### Study subjects

We recruited seven premenopausal and perimenopausal women who fulfilled the American College of Rheumatology criteria for active RA as defined by at least nine tender and six swollen joints, erythrocyte sedimentation rate (ESR) of greater than 28 mm/hour or C-reactive protein (CRP) of greater than 2.0 mg/dl, and morning stiffness of greater than 45 minutes. Use of nonsteroidal anti-inflammatory drugs and/or hydroxychloroquine was permitted. However, drug doses had to have been stable for at least 1 month prior to enrollment and they were held constant during the study unless toxicity required dose reduction. Patients were allowed to be on stable doses of methotrexate, but past use of all other disease-modifying agents (for example, sulfasalazine or cyclosporin) or anti-tumor necrosis factor (TNF) agents (for example, etanercept or infliximab) or glucocorticoid was allowed only if (a) the total exposure had not been more than 3 months and (b) there had been no exposure in the 3 months prior to enrollment. No patients were using alternative treatments such as nutritional supplements, acupuncture, or chiropractic therapy, and all were physically active. At the time of study screening, all patients with RA were either premenopausal, as defined by a history of normal menses and normal estradiol (>30 pg/ml) plus follicle-stimulating hormone (FSH) levels, or perimenopausal, with a history of irregular menses during the 12 months prior to study and normal estradiol (>30 pg/ml) plus elevated FSH (>30 IU/ml) levels. Ten healthy women matched for age (± 3 years), body mass index (BMI) (± 1.0), and menstrual and reproductive hormone status were also included. Research subjects were excluded if they were obese (BMI > 30), had used prescription or over-the-counter estrogen/progesterone preparations during the 2 weeks prior to screening, were pregnant, or had a history of cancer, renal disease, liver disease, anemia, endocrine or metabolic disorders, active infections or live vaccinations (in the 3 months prior to enrollment), depression, or any other comorbid medical or psychiatric condition known to influence the GH-IGF-I or HPA axis. The study was approved by the Institutional Review Board of the National Institute of Diabetes and Digestive and Kidney Diseases and the National Institute of Arthritis and Musculoskeletal and Skin Diseases, National Institutes of Health (NIH), and all participants provided written informed consent.

### Study design

Study participants were admitted to the Clinical Research Center on the evening of day 1 to allow overnight adaptation and provision of their usual *ad libitum *diet in the form of a light dinner. Participants then remained fasting overnight. At 7 a.m. on day 2, an intravenous (IV) catheter was inserted into a forearm vein and was kept open with 0.9% sodium chloride. At 8 a.m., after the overnight fast, 30 ml of blood was collected for measurements of serum IGF-I, IGF-binding protein (IGFBP)-1 and IGFBP-3, glucose, insulin, lipid profile, CRP, and IL-6. From 8 a.m. on day 2 to 8 a.m. on day 3, blood samples (2.5 ml) were collected at 20-minute intervals, and sera were stored at -80°C for subsequent measurements of GH, cortisol, and DHEA. On the morning of day 3, at the completion of the 24-hour frequent blood sampling, the IV catheter was removed, and study participants were asked to complete a visual analog scale for pain and global health. Anthropometric measurements, including body weight, height, and BMI, were obtained, and a dual energy x-ray absorptiometry (DEXA) scan (Hologic QDR 4500; Hologic Inc., Bedford, MA, USA) was performed to assess total and regional lean body mass (LBM), total fat mass, and bone mineral density (BMD) at six sites (postero-anterior spine, total femur, femoral neck, trochanter, Ward's area, and distal radius). Participants were discharged early in the afternoon of day 3.

### Biochemical assays

Serum obtained at 8 a.m. after an overnight fast was used for measurements of IGF-I, IGFBP-1 and IGFBP-3, CRP, and IL-6. GH and cortisol concentrations were measured in sera obtained from the 24-hour (every 20 minutes) sampling technique by means of a chemiluminescence assay (Nichols Institute Diagnostics Inc., San Clemente, CA, USA). Sensitivity and intra- and interassay coefficients of variation (CVs) of the GH assay were 0.1 ng/ml and 2.8% and 7.5%, respectively. Corresponding values for the cortisol assay were 0.9 μg/dl and 4.4% and 11%, respectively. IGF-I, IGFBP-1, and IGFBP-3 were measured at Endocrine Sciences (Tarzana, CA, USA). IGF-I was measured by a blocking radioimmunoassay (RIA) after acid alcohol extraction. IGFBP-1 and IGFBP-3 were measured by RIA in dilute serum. The sensitivity of the IGF-I assay was 63.4 ng/ml, and intra- and inter-assay CVs were 6.5% and 9.4%, respectively. Corresponding values for the IGFBP-1 assay were 5 ng/ml, with intra- and inter-assay CVs of 6% and 12%, and those for the IGFBP-3 assay were 0.8 mg/l, with intra- and inter-assay CVs of 13% and 17%, respectively. Serum levels of DHEA were measured in the every-20-minute sampling specimens by enzyme-linked immunosorbent assay at Diagnostic Systems Laboratories (Webster, TX, USA). The sensitivity of the assay was 0.1 ng/ml, with intra- and interassay CVs of 10.7% and 17.0%, respectively. DHEA measurements correlated strongly (*r*^2 ^= 0.87) with values quantified by tandem gas chromatography-mass spectrometry. IL-6 was measured using commercially available kits (Quantikine HS Human IL-6 Immunoassay; R&D Systems, Inc., Minneapolis, MN, USA). The sensitivity of the IL-6 assay was 0.039 pg/ml with intra- and inter-assay CVs of 7.8% and 7.2%, respectively. Serum concentrations of CRP were measured in the NIH Clinical Center's Department of Laboratory Medicine by high-sensitivity nephelometric assay on an IMMAGE Immunochemistry System (Beckman Coulter, Fullerton, CA, USA). The sensitivity was 0.1 mg/dl and the intra- and interassay CVs were 2.6% and 3.0%, respectively. Serum concentrations of glucose, insulin, total and high-density lipoprotein (HDL) cholesterol, and triglycerides were measured by routine chemical techniques in the NIH Clinical Center's Department of Laboratory Medicine.

### Analysis of hormone secretion

Multi-parameter deconvolution analysis (Deconv) was applied to determine quantitative properties of underlying secretory bursts and endogenous hormone half-life of GH, cortisol, and DHEA [10]. Regularity in GH, cortisol, or DHEA concentration-time series was quantified using approximate entropy (ApEn) as previously described [11].

Twenty-four-hour rhythmicity of serum GH, cortisol, or DHEA concentrations was quantified by cosinor analysis [12]. This procedure entails unweighted regression of a cosine function of 1,440-minute periodicity on the observed hormone concentration-time series. Ninety-five percent statistical confidence intervals are determined for the fitted amplitude (50% of the nadir-zenith difference), mesor (cosine mean), and acrophase (clock time of calculated maximum value).

### Statistical analysis

After verification of lack of difference between women with RA and their healthy controls with respect to age and BMI, an exact Wilcoxon rank sum test was used to compare results between groups. *P *values for the primary outcome measures (total GH level and IGF-I) were adjusted for multiple comparisons by the Hochberg method and considered significant if the *p *value was less than 0.05 [13]. Planned secondary parameters (IGFBPs, IL-6, DHEA, cortisol, and metabolic and lipid parameters) were considered significant if the *p *value was less than 0.01. When unplanned exploratory parameters (DEXA measurements of body fat and BMD) were compared between groups, a *p *value of less than 0.005 was considered significant.

## Results

### Patient characteristics

There were no significant differences in age or BMI between patients with RA and control subjects. Patients with RA were predominantly Hispanic-American and African-American, whereas control subjects were primarily Caucasian and African-American. Patients with RA had experienced their disease for a mean ± standard error of the mean of 7.6 ± 2.6 years, had 23.2 ± 3.7 swollen joints and 23.3 ± 2.8 tender joints, had increased pain and physical component summary scores, and exhibited elevated values for ESR, CRP, and IL-6 (Table 1).

**Table 1:** Characteristics of patients with rheumatoid arthritis

Variables	Rheumatoid arthritis (*n *= 7)	Controls (*n *= 10)
Age (years)	36.3 ± 3.7	41.8 ± 2.1
Race (*n*)		
Caucasian	0	6
Hispanic	5	0
African-American	2	4
Duration of rheumatoid arthritis (years)	7.6 ± 2.6	NA
Swollen joint count^a^	23.2 ± 3.7	NA
Tender joint count^a^	23.3 ± 2.8	NA
Swollen joint score^a^	31.7 ± 6.1	NA
Tender joint score^a^	34.0 ± 5.5	NA
Pain (visual analog) scores^b^	7.1 ± 0.6	NA
Physical component summary score^c^	28.0 ± 2.1	55.3 ± 1.3
Erythrocyte sedimentation rate (mm/hour)	69.1 ± 11.3	15.9 ± 1.6
C-reactive protein (mg/dl)	3.7 ± 1.3	0.4 ± 0.02
Interleukin-6 (pg/ml)	25.6 ± 9.8	5.3 ± 2.4

Values are presented as mean ± standard error of the mean. NA, not applicable. ^a^Sixty-eight joints were examined for tenderness, and 66 joints were examined for swelling; ^b^visual analog scale (cm) ranged from 0 (best) to 10 (worst); ^c^values represent percentile scores.

### Hormone measures

Except for a shorter GH half-life in patients with RA, there were no significant differences in circadian GH deconvolution parameters or ApEn scores in patients with RA versus control subjects (Table 2). However, in RA patients as compared with their healthy counterparts, the mean serum concentration of IGF-I in the morning was lower (*p *= 0.05), IGFBP-3 exhibited a trend toward being lower, and IGFBP-1 concentrations tended to be higher. The changes in IGF-I and IGFBPs in patients with RA were associated with nonsignificantly higher 24-hour total GH production rates.

**Table 2:** GH secretory parameters and morning serum concentrations of IGF-I and IGFBPs in rheumatoid arthritis patients and control subjects

Variables	Rheumatoid arthritis (*n *= 7)	Controls (*n *= 10)	*P *value
GH basal secretion (μg/liter per minute)	0.02 ± 0.00	0.01 ± 0.00	NS
GH mass/burst (μg/liter)	6.12 ± 1.03	6.71 ± 1.20	NS
GH burst frequency (number/24 hours)	13.7 ± 1.9	9.8 ± 1.2	NS
GH amplitude (μg/liter per minute)	0.430 ± 0.10	0.29 ± 0.06	NS
GH total production rate (μg/liter per 24 hours)	114 ± 26	73 ± 74	0.28
Mean GH (μg/liter)	0.91 ± 0.14	1.20 ± 0.17	NS
Integrated GH (μg/liter per minute)	1,316 ± 201	1,717 ± 245	NS
GH half-life (minutes)	9.2 ± 1.2	14.6 ± 0.9	0.0037
GH approximate entropy	0.75 ± 0.07	0.70 ± 0.09	NS
IGF-I (ng/ml)	129 ± 27	205 ± 25	0.05
IGFBP-1 (ng/ml)	42.1 ± 16	8.3 ± 2	0.066
IGFBP-3 (ng/ml)	2.2 ± 0.2	2.6 ± 0.1	0.058

Values are presented as mean ± standard error. *P *value indicates the significance of the difference in each parameter value between patients and control subjects. See 'Statistical analysis' section for details. GH, growth hormone; IGF-I, insulin-like growth factor-1; IGFBP, insulin-like growth factor-binding protein; NS, not significant (*p *> 0.10 when not explicitly reported).

We also examined the circadian characteristics of cortisol and DHEA secretion. There were no significant differences in the total production rate, mean or integrated concentrations, or regularity (ApEn) of circadian cortisol or DHEA secretion in RA patients compared with healthy control subjects (Table 3). The amplitudes and acrophases of 24-hour cortisol or DHEA rhythms were also similar in the two groups (data not shown).

**Table 3:** Diurnal cortisol and dehydroepiandrosterone secretory parameters in rheumatoid arthritis patients and control subjects

	Cortisol (μg/dl)		Dehydroepiandrosterone (ng/ml)	
Variables	Rheumatoid arthritis (*n *= 7)	Controls (*n *= 10)	Rheumatoid arthritis (*n *= 7)	Controls (*n *= 10)

Total production rate (per 24 hours)	71 ± 8	75 ± 7	280 ± 108	203 ± 50
Mean concentration	7.12 ± 0.62	6.48 ± 0.33	7.02 ± 2.28	6.76 ± 0.89
Integrated concentration (per minute)	10,094 ± 841	9,218 ± 598	9,950 ± 3,252	9,401 ± 1,275
Approximate entropy	0.93 ± 0.08	0.91 ± 0.08	1.22 ± 0.08	1.18 ± 0.06

Values are presented as mean ± standard error. All of the differences had *p *values greater than 0.10.

### Body composition and metabolic profile

Total LBM, determined by DEXA, was lower in patients with RA versus control subjects, with disproportionately greater reductions in the legs versus the arms (Table 4). In contrast, there were no significant group differences in absolute or percentage total fat mass or in BMD values of the spine, hip, or radius (Table 4). After an overnight fast, serum creatinine and (to a lesser extent) total cholesterol concentrations were lower, whereas serum insulin concentrations were slightly but nonsignificantly higher in patients with RA; there were no group differences in glucose, QUICKI (Quantitative Insulin Sensitivity Check Index), low-density lipoprotein or HDL cholesterol, or triglyceride values.

**Table 4:** Body composition and metabolic outcomes in rheumatoid arthritis patients and control subjects

Variables	Rheumatoid arthritis (*n *= 7)	Controls (*n *= 10)	*P *value
Body mass index (kg/m^2^)	26.9 ± 0.9	27.8 ± 0.9	NS
Total lean body mass (kg)	40.3 ± 1.1	46.1 ± 1.6	0.019
Lean body mass, both arms (kg)	3.9 ± 0.3	4.6 ± 0.2	0.03
Lean body mass, both legs (kg)	12.5 ± 0.4	15.4 ± 0.7	0.01
Total body fat mass (kg)	23.7 ± 0.8	25.8 ± 1.5	NS
Body fat (percentage)	35.9 ± 1.1	34.6 ± 1.0	NS
Bone mineral density (g/cm^2^)^a^			
Femoral neck	0.85 ± 0.03	0.82 ± 0.05	NS
Trochanter	0.69 ± 0.03	0.75 ± 0.05	NS
Ward's area	0.74 ± 0.03	0.74 ± 0.06	NS
Lumbar spine (L2-L4)	0.790 ± 0.03	0.81 ± 0.04	NS
Distal radius	0.68 ± 0.01	0.72 ± 0.02	NS
Serum creatinine (mg/dl)	0.57 ± 0.05	0.79 ± 0.02	<0.001
Fasting blood glucose (mg/dl)	90.6 ± 1.9	91.7 ± 2.2	NS
Fasting insulin (μU/liter)	11.20 ± 3.60	7.40 ± 0.70	NS
Quantitative Insulin Sensitivity Check Index	0.35 ± 0.01	0.36 ± 0.01	NS
Total cholesterol (mg/dl)	156 ± 13	198 ± 13	0.046
Low-density lipoprotein cholesterol (mg/dl)	96.6 ± 11.2	124.4 ± 13.3	NS
High-density lipoprotein cholesterol (mg/dl)	47.90 ± 5.30	57.7 ± 4.2	NS
Triglycerides (mg/dl)	97.6 ± 9.2	109.3 ± 11.5	NS

Values are presented as mean ± standard error. *P *value indicates the significance of the difference in each parameter value between patients and control subjects. ^a^One patient with rheumatoid arthritis was removed from the bone mineral density analysis because of her diagnosis of osteosclerosis. NS, not significant (*p *> 0.10).

## Discussion

In this study, a well-characterized group of pre- and perimenopausal women with clinically and biochemically active RA, compared with age- and BMI-matched healthy women, exhibited reduced morning serum concentrations of IGF-I, a trend toward lower IGFBP-3, accelerated GH circulatory half-life, trends toward increases in IGFBP-1 and IL-6 levels (and total GH production), unaltered pulsatile, nycthemeral, or feedback-sensitive (entropic) features of cortisol or DHEA secretion, and substantially decreased LBM, especially in the legs.

Studies evaluating the GH-IGF-I-IGFBP-3 system in patients with RA have yielded contradictory and inconsistent results [7,9,13-18], in part because of differences in the ages, genders, and numbers of patients studied, disease activity, and use of glucocorticoids and other disease-modifying agents. In the current investigation, the mean serum concentration of IGF-I in the morning was lower in patients with RA versus control subjects and IGFBP-3 also exhibited a similar trend, findings consistent with some studies [15,16] but not with others [18,19]. In the latter four studies, there were no apparent relationships between disease activity and IGF-I and IGFBP-3 levels. Most circulating IGF-I is produced by the liver in response to GH and mediates many of the anabolic actions of GH. In comparison, local IGF-I production within target tissues is regulated by both GH-dependent and -independent mechanisms. IGF-I circulates as a ternary complex with IGFBP-3 and the acid-labile subunit, and both liver-derived proteins are under the control of GH [20]. Reduced serum concentrations of IGFBP-3 can result from a primary decrease in IGFBP-3 production, or secondarily, due to a reduction in IGF-I. IGFBP-3 stabilizes circulating IGF-I, and reductions in IGFBP-3 can contribute to a decrease in IGF-I levels (due to decreased stability of the complex). IGFBP-1, which is also derived from the liver, binds to free IGF-I and is negatively regulated by nutrition and insulin [20]. Elevated IGFBP-1 levels as observed in patients with RA could further reduce free IGF-I availability and action [21].

In this study, mean GH concentrations in patients with RA were not significantly different from those in age- and BMI-matched healthy volunteers. However, the nonsignificant increase in GH total production rate which we observed was accompanied by a significant reduction in the calculated GH half-life in patients with RA, and that may explain the unchanged mean and integrated circulating GH concentrations. Circulating GH is cleared primarily by the liver and kidney. The rate of GH elimination is directly related to the plasma total free GH concentration, relative obesity, and renal function [22]. The exact mechanism (or mechanisms) of the reduced GH half-life in our patients with RA is unclear, and the apparent change in calculated GH elimination kinetics in patients with RA requires further confirmation by more robust, isotopic infusion techniques. However, some potential factors may explain the reduced GH half-life in our patients with RA. GH is catabolised in the kidney after filtration and absorption by the proximal tubules. Consequently, GH clearance rate is determined by the glomerular filtration rate (GFR). In this study, estimated GFR (using the LBM-adjusted Cockcroft and Gault formula or the formula derived from the Modification of Diet in Renal Disease [MDRD] study [23,24]) was higher in RA patients as compared with healthy volunteers (MDRD-derived GFR: 123.9 ± 14.2 versus 77.2 ± 8.1 ml/minute per 1.73 m^2^; *p *= 0.0015). This may have contributed to the shortened GH half-life. In addition, GH half-life is determined by the volume of distribution. In this study, LBM is significantly reduced in RA patients as compared with healthy controls. Consequently, it is possible that the volume of distribution for GH is also reduced. Thus, increased renal clearance and reduced volume of distribution may enhance GH elimination. Of note, renal impairment in RA occurs late in the course of disease and is increased in patients who develop vasculitis or amyloidosis or as a complication from drug therapy. The most potentially nephrotoxic agents – gold salts, penicillamine, and cyclosporine – are no longer commonly used. Thus, the finding of reduced GH half-life observed in this study may be more prominent in earlier disease and in patients who have not received long-term disease-modifying anti-rheumatic drug therapy.

The pattern of reduced circulating IGF-I and IGFBP-3 with an unchanged GH total production rate in patients with RA, as observed in our study, appears to be consistent with GH resistance or insensitivity [20,25]. Although the exact mechanisms for GH insensitivity in patients with RA are unclear, GH resistance has been observed in inflammatory and heightened catabolic states [26]. Cytokine exposure (IL-1, TNF-α, and endotoxin) in animals decreases IGF-I synthesis [27,28], and reduced IGF-I levels occur in patients with chronic liver disease [29] and in critically ill patients [26]. Similarly, cytokines upregulate IGFBP-1 synthesis [30]. Of note is the recent report by Nemet and colleagues [31] demonstrating that short-term infusion of recombinant human IL-6 in healthy young men to levels typically occurring during exercise decreases serum concentrations of IGF-I and increases those of GH and IGFBP-1. Our findings of increased inflammatory markers (ESR, CRP, and IL-6), along with reduced IGF-I and IGFBP-3, are consistent with data from some but not all prior studies. Rall and colleagues [19] found no alterations in GH kinetics (frequent sampling followed by deconvolution) in RA patients compared with age- and BMI-matched control subjects. However, the authors did observe a trend toward reduced IGF-I concentrations in the patients with RA (*P *= 0.08). In the latter study, data from male and female patients with RA were evaluated together, patients had a longer duration of RA, and they were on stable doses of prednisone and/or methotrexate – all of which may have confounded the authors' observations. Other studies have measured GH secretion after stimulation with GH-releasing hormone [7] or insulin-induced hypoglycemia [9,17] rather than assessing spontaneous, diurnal GH secretion (as in this study), rendering any comparisons and subsequent conclusions between the studies difficult. In another study, GH concentrations in single morning (8 a.m.) samples were elevated approximately fivefold in RA patients taking glucocorticoids as compared with values in healthy controls, whereas IGF-I and IGFBP-3 levels were similar in the two groups [18]. Although we are unaware of reports in which IGF-I and IGFBP responses to exogenous GH have been compared in patients with RA and healthy control subjects, the present study and other studies suggest that RA is associated with GH resistance or insensitivity.

The effects of RA *per se *on the HPA axis have been reported in multiple studies [8,9,32]. To date, there has been no consistent demonstration of altered basal or stimulated cortisol production in RA patients as compared with healthy individuals [32]. However, the presence of 'normal' cortisol levels in the face of increased secretion of cytokines (IL-6) has been a consistent finding, leading some to suggest that RA is characterized by a state of 'relative hypocortisolism,' with an inadequate anti-inflammatory response to inflammation [32-34]. In our study, patients with RA exhibited elevated morning ESR, CRP, and IL-6 concentrations but had no alteration in pulsatile, nycthemeral, or entropic features of spontaneous cortisol secretion. Diminished adrenal androgens have been reported in premenopausal women with RA [35-37]. In these studies, dehydroepiandrosterone sulfate (DHEAS) and to a lesser extent DHEA, concentrations in single morning samples were lower in patients with RA. Additionally, Cutolo and colleagues [38] reported that morning DHEA levels were inversely related to the ESR and that the DHEA response to adrenocorticotropic hormone (ACTH) stimulation was decreased in premenopausal women with RA. To our knowledge, the current study is the first to report spontaneous, diurnal DHEA secretion in patients with active RA. Prior findings of diminished DHEAS levels, coupled with our observation of unaltered circadian DHEA secretion in patients with RA, might be explained in part by a decreased conversion of DHEAS to DHEA resulting from excess proinflammatory cytokines, as has been reported in synovial fluid from patients with RA [37]. Moreover, our DHEA findings further suggest that in the setting of heightened inflammatory and cytokine burden, there is a relative adrenocortical androgen insufficiency in patients with RA. In support of this view, neutralization of IL-6 increases androgen secretion in patients with RA [39].

Glucocorticoids exert negative feedback control on the HPA axis by suppressing hypothalamic CRH production and ACTH secretion. The time required to achieve suppression and recovery is variable and is dependent upon the route, dosage, duration, and dosing schedule [40]. Due to suppressive effects of corticosteroid use in patients with RA, we cannot entirely rule out persistent impairment of HPA activity. Four of the patients with RA had taken glucocorticoids, with a cumulative exposure in each that was not more than 3 months, and all patients with RA had been off steroids for at least 3 months prior to study enrollment. In addition, there were no differences in early a.m. or peak plasma concentrations of cortisol, ACTH, or DHEA. Adrenal androgen secretion is more sensitive than cortisol production to the suppressive effects of glucocorticoid therapy [41]. In this study, basal and peak DHEA levels are unchanged in RA patients as compared with healthy individuals. Moreover, IL-6 is known to stimulate cortisol and androgen production in an ACTH-independent fashion [39,42]. These findings, in concert with the relatively short duration of past glucocorticoid therapy, suggest that the normal levels of cortisol in patients with RA in this study are less likely (but cannot be ruled out entirely) due to an impaired HPA axis by prior steroid use.

Our patients with RA exhibited reduced LBM, consistent with findings in other studies [1,19]. The decrease in lean mass was especially evident in the legs and was accompanied by diminished serum concentrations of creatinine, an established index of skeletal muscle mass. Cachexia, characterized by the loss of body cell mass and function, frequently occurs in patients with RA. Relative hyposomatotropism, due to reduced activity of GH/IGF-I axis and the associated negative anabolic balance resulting from abnormalities in cytokine, cortisol, and adrenal steroid production and action, have been proposed to play significant roles in rheumatoid cachexia [4].

Several limitations of this study deserve comment. Because of strict inclusion and exclusion criteria, the accrual of patients with RA was below the intended number of subjects planned. Consequently, we consider any results of interest to be hypothesis-generating, in that they require confirmation in an independent, larger group of patients. Additionally, the relative homogeneity of our study population does not allow for extrapolation of our findings to postmenopausal women or men. Finally, quality and quantity of sleep were not measured, and their possible influences on circadian rhythms of the hormones measured could not be ascertained.

## Conclusion

The current study suggests that active RA in pre- and perimenopausal women is characterized by a state of relative GH insensitivity and diminution in diurnal cortisol and DHEA secretion, given the chronic inflammatory state of the patients. Whether these combined somatotropic and adrenocortical abnormalities in a proinflammatory cytokine milieu exacerbate the inflammatory process and play a role in the pathogenesis of rheumatoid cachexia remains to be determined.

## Abbreviations

ACTH = adrenocorticotropic hormone; ApEn = approximate entropy; BMD = bone mineral density; BMI = body mass index; CRH = corticotropin-releasing hormone; CRP = C-reactive protein; CV = coefficient of variation; DEXA = dual energy x-ray absorptiometry; DHEA = dehydroepiandrosterone; ESR = erythrocyte sedimentation rate; FSH = follicle-stimulating hormone; GFR = glomerular filtration rate; GH = growth hormone; HDL = high-density lipoprotein; HPA = hypothalamic-pituitary-adrenal; IGF-I = insulin-like growth factor-1; IGFBP = insulin-like growth factor-binding protein; IL-6 = interleukin-6; IV = intravenous; LBM = lean body mass; MDRD = Modification of Diet in Renal Disease; NIH = National Institutes of Health; RA = rheumatoid arthritis; RIA = radioimmunoassay; TNF = tumor necrosis factor.

## Competing interests

The authors declare that they have no competing interests.

## Authors' contributions

MRB participated in all aspects of conceptualization, design, implementation, and data analysis and in drafting this manuscript. RM participated in data analysis and in drafting this manuscript. MW and BEM participated in all patient recruiting and management. HLB and KAW participated in patient recruiting and overnight sampling studies and performed the GH, cortisol, and DHEA assays. CS, MM, and SA participated in patient recruitment and data collection and management. JR performed and interpreted the DEXA scans. SMS contributed to the study design and performed all statistical analyses. RG-M participated in all aspects of conceptualization, design, implementation, data analysis and in writing this manuscript. All authors read and approved the final manuscript.
